# Growth Performance, Rumen Fermentation, In Vivo Digestibility, and Meat Quality of Pelibuey Lambs Fed a Diet with Ensiled Coffee Pulp

**DOI:** 10.3390/ani13223462

**Published:** 2023-11-09

**Authors:** Graciela Munguía-Ameca, María Esther Ortega-Cerrilla, José Guadalupe Herrera-Haro, Ricardo Bárcena-Gama, Cuauhtémoc Nava-Cuéllar, Pedro Zetina-Córdoba

**Affiliations:** 1 Programa de Recursos Genéticos y Productividad-Ganadería, Colegio de Postgraduados, Campus Montecillo, Montecillo 56230, Estado de México, Mexico; gamun@hotmail.com (G.M.-A.); haro@colpos.mx (J.G.H.-H.); rbarcena@colpos.mx (R.B.-G.); 2 Facultad de Medicina Veterinaria y Zootecnia, Universidad Nacional Autónoma de México, Ciudad de México 04510, Mexico; cnava@unam.mx; 3 Programa de Ingeniería Agroindustrial, Universidad Politécnica de Huatusco, Huatusco de Chicuéllar 94100, Veracruz, Mexico

**Keywords:** meat quality, antioxidant capacity, ensiled coffee pulp

## Abstract

**Simple Summary:**

This research focused on the potential use of coffee pulp, a byproduct of agriculture, in the diet of Pelibuey lambs. Although caffeine and tannin content limit its use in animal feeding, the study evaluated the inclusion of ensiled coffee pulp in the lambs’ diet. The research examined antioxidant compounds, caffeine, and tannin content in the lambs’ diets; their productivity, ruminal fermentation variables, in vivo digestibility, antioxidant capacity in blood serum; and carcass and meat characteristics. The study found that adding the ensiled coffee pulp to the diet did not affect caffeine and tannin concentration. However, it did improve antioxidant capacity in the blood serum, crude protein digestibility, and a slight increase in the redness (a*) of the meat. The research suggests that using up to 20% of ensiled coffee pulp in Pelibuey lambs’ diets is feasible without affecting their productivity, ruminal fermentation variables, nutrient digestibility, or carcass and meat characteristics.

**Abstract:**

Coffee pulp has been included in ruminant diets; but until now, little has been known about how the addition of ensiled coffee pulp (ECP) affects the growth performance of lambs. This study explores the diet’s antioxidant capacity, tannins, and caffeine concentration and its effect on water intake, growth performance, rumen variables, in vivo digestibility, nitrogen balance, and carcass and meat characteristics of lambs fed ECP. Thirty-six male Pelibuey lambs were distributed randomly to one of three treatments (*n* = 12): without ECP_0_; diet with 10% ECP_10_, and diet with 20% ECP_20_. The diets’ antioxidant capacity, tannins, and caffeine concentration were similar (*p* > 0.05) for all treatments. The diets’ antioxidant compounds and the blood serum’s antioxidant capacity were affected (*p* < 0.05). Dry matter and water intake, body-weight gain, and feed conversion were not significant (*p* > 0.05). No differences (*p* > 0.05) were found in the rumen variables or the nitrogen balance. However, the in vivo digestibility of crude protein was affected (*p* < 0.05). Carcass and meat quality were not affected (*p* > 0.05) by the inclusion of ECP, except temperature and rednes*s* (a*) at seven days of storage, respectively. Including up to 20% of ECP in the diet of lambs did not affect the growth performance, rumen variables, or nitrogen balance; however, the antioxidant compounds of the diets, the antioxidant capacity in blood serum, and the in vivo digestibility of crude protein were different. There was an increase in the redness (a*) and lower temperature in the *Longissimus dorsi* muscle, keeping lightness (L*), yellowness (b*), water-holding capacity, and texture at seven storage days.

## 1. Introduction

A residue or byproduct (pulp, silver skin, and parchment) is generated during coffee processing, leading to severe environmental harm [1]. This coffee pulp contains disproportionate amounts of phenolic compounds and caffeine [2]. Among the most important benefits of phenolic compounds and caffeine are the inhibition of fatty acid peroxidation [3] and the protection of the liver, as well as their hypoglycemic, antiviral, and antibacterial functions [4]. All these benefits may favor carcass characteristics by preventing or decreasing oxidation. Among the phenolic compounds, tannins are the most significant due to their abundance in several plant tissues, including the seed coat. Although caffeine has been considered antinutritional because it reduces acceptability and palatability [5], today, it is desirable in diets and feed due to its antioxidant capacity [6]. Patra and Yu [7] stated that the moderate use of forage that contains tannins reduces protein degradation in the rumen and improves body weight, wool growth, milk production, and reproductive yield. Coffee pulp is only available for three to four months during the year. It has high water content and antinutritional factors that limit its use in the diet [8]. Researchers have included coffee pulp in sheep diets in varying percentages, 7, 8, 14, 16, 21, and 28% to evaluate productive performance [9,10] or 5% and 10% to investigate reproductive parameters [11]. In cows, coffee pulp was supplemented at 12% of their diet, approximately 1.2 kg per day, to evaluate milk production [12,13]. Additionally, coffee pulp has been used in juvenile Tilapia at a rate of up to 18% [14]. However, little has been known about the effect of the addition of ensiled coffee pulp on the productive performance and carcass and meat characteristics of several domestic species. Including 16 and 28% ensiled coffee pulp in the diets of lambs does not affect the productive lamb parameters [9,10]. Therefore, this study assesses the antioxidant capacity, antioxidant compounds, tannin, and caffeine concentration of diets that included 0, 10, and 20% ensiled coffee pulp (ECP) and the effects on growth performance, rumen variables, in vivo digestibility, nitrogen retention, carcass, and meat characteristic when fed to Pelibuey lambs.

## 2. Materials and Methods

### 2.1. Localization and Ethical Manifestation

The study was carried out at the Colegio de Postgraduados, Campus Montecillo, Mexico (19°29′ W, 98°53′ N), from August to November. All experimental procedures with the sheep were approved by the Animal Welfare Committee of the Colegio de Postgraduados, according to regulations established by the Animal Protection Law enacted by the State of México.

### 2.2. Animals, Treatments, and Experimental Design

Thirty-six weaned male Pelibuey lambs with an initial average body weight of 20.4 ± 2.59 kg were utilized. The animals were dewormed (1 mL/50 kg PV, Ivomec^®^ F, Boehringer Ingelheim, Ingelheim am Rhein, Germany), vitamins ADE (2 mL/lamb, Vigantol^®^ ADE, Bayer, Mexico City, Mexico) and 3 mL of metabolic stimulant (3 mL/lamb, Catosal^®^, Bayer, Mexico City, Mexico) were intramuscularly injected, and housed in individual pens with ad libitum access to feed and water. In a completely random design, animals were randomly assigned to one of three treatments (*n* = 12): control diet, without ECP (ECP_0_) and diet with 10% (ECP_10_) and 20% (ECP_20_) of ECP, respectively. The lambs were adapted to the diet for 20 days and fattened for 60 days. The composition of the diets (Table 1) was formulated according to NRC [15]. The coffee pulp was obtained in Huatusco, Veracruz, Mexico, from a depulping plant that uses the wet method to remove the berries from the coffee bean. The coffee pulp was drained for 12 h to eliminate any water it absorbed during the depulping process; later, it was ensiled for 140 days, and, immediately after opening the silo, it was dehydrated in the sun for 8 days. The ECP contained 13.24% crude protein (CP), 10.82% ash, 1.72 ether extract (EE), 31.25% lignin, 55.10% neutral detergent fiber (NDF), 52.14% acid detergent fiber (ADF), tannins (1.189 mg/g dry matter), and three antioxidants in more significant proportion, namely ferulic acid (4.256 mg/g of dry matter), caffeic acid (4.913 mg/g of dry matter), and chlorogenic acid (4.875 mg/g of dry matter).

### 2.3. Performance, Backfat Thickness, Longissims Dorsi Area, and Blood Sampling

The lambs were weighed at the beginning of the experiment and then every 15 days to determine their daily weight gain (DWG), obtained by the difference in body weight (kg) divided by the number of days between weights. Daily dry matter intake (DMI) was estimated individually by the difference in the weight (kg) between the offered feed and that refused. Feed conversion (FC) was calculated individually from the ratio DMI/DWG for each weighing period. Daily water intake (WI) was measured using a 1 L receptacle graduated in milliliters.

At 0, 30, and 60 days of the experiment, the backfat thickness was measured in all the lambs and the LD area using an ultrasound (Model SV-600, Medison Sonovet 600^®^, Inc., Cypress, CA, USA) with a linear transducer of 7.5 MHz in a perpendicular position to the dorsal median line between the 12th and 13th ribs [16]. Lectures were obtained in square millimeters for the ribeye area and centimeters for backfat thickness [17].

Blood samples were taken at the beginning and end of the experiment [9]. Five milliliters were individually collected from the jugular vein into vacutainer tubes with three milliliters EDTA. Serum was obtained by centrifugation (Model IEC Centra CL8R centrifuge, Thermo Electron Corporation^®^, Waltham, MA, USA) at 1957× *g* for 10 min at 4 °C, and the plasma obtained was kept at −20 °C in a freezer (Model EUR251P7W, Tappan, Electrolux Home Products North America, Charlotte, NC, USA) until analysis.

### 2.4. pH, Ammonia Nitrogen, Volatile Fatty Acid, and Total Ruminal Bacteria and Protozoa

Samples of ruminal liquid (20 mL) were taken from the median ventral part of the rumen by an esophageal probe, 3 h after the morning feeding, from eight lambs in each treatment on days 0 and 60 of the experimental phase; the pH was immediately measured with a portable pH meter (Model Orion 3-Star, Thermo Scientific^®^, Chelmsford, MA, USA). The rumen liquor samples were acidified with metaphosphoric acid (25%) at a 4:1 ratio. The NH_3_-N concentration was determined through absorbance in an ultraviolet light spectrophotometer (Model Cary 1E, Varian^®^, Santa Clara, CA, USA) at 630 nm, according to McCullough (1967). The VFA concentration acetic (AcetA), propionic (PropA), and butyric (ButA) acids were determined according to Erwin et al. [18] using a gas chromatograph (Model Clarus 500, Perking Elmer^®^, Shelton, CT, USA) with an FFAP Elite capillary column, hydrogen as the carrier gas, with a flow of 5.5 mL/min, and with the injector set at 250 °C.

The total ruminal bacteria were quantified in ruminal liquid samples obtained at 60 days of the experiment. Four ml of ruminal liquid were mixed with 1 mL 10% formaldehyde, observing the mix in a Petroff–Hausser chamber. The count was performed following the method described by SIGMA (1990) [19]. The concentration was calculated with the following equation: total bacteria/mL = (average) (dilution factor) (2 × 10^7^). Total protozoa were determined using a Neubauer chamber, a microscope (Model BX51, Olympus, Tokyo, Japan), and the following equation: total protozoa/mL = (average) (dilution factor) (10^4^) to obtain the concentration [20].

### 2.5. In Vivo Digestibility and Nitrogen Balance

After 60 days of the productive performance test, the in vivo digestibility of dry matter (DM), organic matter (OM), CP, NDF, and ADF was measured in five lambs. Animals were selected randomly from each treatment. Feces collection bags were attached for 12 days; during the first 4 days of the adaptation period, the feed intake was adjusted to 90% of that consumed ad libitum. For another 8 days, the feces were collected in the morning and weighed, with 100 g samples being taken daily from each animal and stored at 4 °C. At the end of the digestibility trial, the daily samples were mixed to obtain an individual sample for analysis; in vivo digestibility was determined as the difference between the nutrient ingested and excreted. Digestibility was calculated with the Harris equation [21]. Two days before the end of the feces collection, urine was also collected using buckets placed under the metabolic pens, the volume was recorded, and urine samples were acidified with hydrochloric acid (50%) 50 mL/L. The nitrogen balance was estimated as the difference between the ingested nitrogen and nitrogen excreted, using the nitrogen values of feed, feces, and urine [21].

### 2.6. Carcass Characteristics and Meat Chemical Composition

At the end of the in vivo digestibility test, after fasting for 12 h, the lambs were slaughtered in a commercial slaughterhouse according to NOM-033-SAG/ZOO-2014 [22] (methods of slaughter for both domestic and wild animals). Immediately, the rumen and intestines were weighed with and without gastrointestinal content. The same was done for mesenteric fat and viscera. Carcass length was measured according to De Boer et al. [23]. Hot carcass weight (HCW) was determined; also, cold carcass weight (CCW) was determined after cooling at 5 °C for 24 h; the hot and cold carcass yields were calculated by dividing HCW or CCW by slaughter weight × 100 [24]. pH and temperature were measured with a portable potentiometer (Model HI 99161, Meat HANNA^®^, Waterproof Tester, Woonsocket, RI, USA) with a digital liquid penetration electrode in the *Longissimus dorsi* muscle (LD) between the last thoracic vertebra and first lumbar vertebra both in the hot and cold carcass [25]. Dry matter, crude protein, and ash were determined in the LD frozen at −4 °C for seven days, according to AOAC [26].

### 2.7. Physicochemical Characteristics of the Meat

A meat quality evaluation was done on LD muscle. Samples were frozen after slaughter for 24 h (5 °C) and 7 d (−4 °C) [26]. pH and temperature lectures were performed in triplicate for each sample. A potentiometer (Model HI 99161, Meat HANNA^®^, Waterproof Tester, Woonsocket, RI, USA), was used to measure the pH. For meat color evaluation, a portable colorimeter (Model CR-400/410), Konica Minolta^®^, Ramsey, NJ, USA) L* (lightness), a* (red to green), and b* (yellow to blue) were measured according to CIE-L* a* b* guidelines for meat color measurement [27]. LD muscle samples were exposed to the environment for 20 min for color stabilization. Final values were reported as the mean of 3 measurements in the muscle surface. Water-holding capacity (WHC) was determined by homogenizing 5 g of minced meat in centrifuge tubes with a glass rod for one min with a solution of 8 mL 0.6 M of NaCl. The tubes were immediately deposited in an ice bath for 30 min and shaken for another min. Finally, the tubes were centrifugated (Model 420101, Clay Adams^®^, Sparks, NV, USA) at 1957× *g* 15 min. The supernatant was decanted in a test tube to determine the unretained water [25]. Tenderness was determined using the Warner Bratzler shear blade in a meta texture analyzer (Model TA-XT2, Texture Technologies Corp., Scarsdale, NY, USA) with a speed of 5 mm/s, a break distance of 40 mm, and meat cutting force of 0.918 N in 2 s. The samples were cut as cubes of 1 cm^3^ and placed transversely to the blade’s edge [25].

### 2.8. Chemical Analysis

The diets (DM, OM, CP, EE, and Ash), feces (DM, OM, and CP), and meat (moisture, DM, CP, and ash) were analyzed according to the AOAC (1990). In the diets, NDF and ADF were determined by Van Soest et al. [28]. Feces and urine samples were analyzed for nitrogen (N) [29]. The antioxidant capacity of the diets and its effect on blood serum and the meat was determined by the FRAP (ferric reducing antioxidant power) technique of Benzie and Strain [30], with some modifications [9]. The aldehydes, which are used as a marker of lipid peroxidation, were assessed by measurements of thiobarbituric acid-reactive substances (TBARS) [31] and expressed as malondialdehyde equivalents (MDA, nmol/mg protein). They were determined using the technique of Ohkawa et al. [32]. Phenolic acids were assessed using high-performance liquid chromatography (Model 1100 Series, Agilent Technologies^®^, Santa Clara, CA, USA) with a photodiode array detector and a Nucleosil column Model Nautilus (100-5 C18, 125 × 4 mm), with HPLC grade H_2_O in channel A. Furthermore, the pH was 2.5 after being adjusted using trifluoroacetic acid and acetonitrile in channel B. The gradient was as follows: min 10, A: 85%, B: 15%; min 20, A: 65%, B: 35%; and min 23, A: 65%, B: 35%. The flow rate was one ml per min at 30 °C; 20 μL of the sample was injected, and the reading was taken at 280 nm. The phenolic acid standards were Sigma (e.g., gallic, chlorogenic, syringic, vanillic, 2,5-di-hydroxybenzoic, caffeic, p-hydroxybenzoic, 2,3-dihydroxybenzoic, ferulic, and p-coumaric acids). Caffeine was quantified by isocratic analyses, using water and HPLC grade acetonitrile in a 75:25 proportion with a Nucleosil column (similar to that used for phenolic acid determination), in which 10 µL of the sample were injected at a flow rate of 0.8 mL per min at 25 °C, and reading occurred at 273 nm. Caffeine (Merck^®^, Darmstadt, Germany) was used at concentrations of 2 to 12 µg to generate a standard curve with 5 points [9].

Tannins were analyzed in three phases. In the first stage, 10 mL of 70% acetone were added to a 25 mg sample, placed in an ultrasound bath (Model B220, Branson Ultrasonic^®^, Apeldoorn, The Netherlands) for 10 min, rested for 5 min, and then subjected to sonication for 10 min. Samples were immediately centrifuged at 1957× *g* for 10 min. The supernatant was collected and placed on ice. In the second stage, one milliliter of distilled H_2_O was added to 100 mg polyvinyl pyrrolidone and 1 mL of the extract with tannins. This mixture was shaken and stored at 4 °C for 15 min, shaken for 1 min, and centrifuged at 1409× *g* for 5 min. The supernatant was collected and placed on ice. In the third stage, a standard curve was obtained with 0, 2, 4, 6, 8, and 10 μg solutions of tannic acid prepared from a mother solution (0.1 mg/mL), distilled H_2_O (500, 480, 460, 440, 420, and 400 µL), Folin’s reagent (250 µL), and 20% sodium carbonate (1.250 mL). The mixture was measured in a spectrophotometer (Model Cary 1E, Varian^®^, Santa Clara, CA, USA) at an absorbance wavelength of 725 nm.

### 2.9. Statistical Analyses

Data were analyzed as a completely randomized design with SAS [33], and the means were compared with the Tukey test. Data on caffeine, tannin, antioxidant compounds, antioxidant capacity, digestibility, nitrogen balance, total ruminal bacteria, protozoa, characteristics of the carcass, and chemical composition of the meat were analyzed using PROC GLM, according to the following model (Equation (1)):

Y*ij* = µ + τ*i* + Є*ij* (1)

where Y*ij* is the *j*th observation in the *i*th treatment, µ is the general mean, τ*i* is the effect of the *i*th treatment, and Є*ij* is the random error.

Data on the DWG, DMI, FC, WI, ruminal pH, VFA, and NH_3_-N, characteristics, and antioxidant activity of meat were analyzed as repeated measures using the PROC MIXED, according to the following model (Equation (2)):

Y*ijk* = µ + τ*i* + δ*j*(*i*) + P*k*+ (τP)*ik* + Є*ijk* (2)

where Y*ijk* is the response variable, µ is the general mean, τ*i* is the effect of the *i*th treatment, δ*j*(*i*) is the effect of the *j*th replicate within the *i*th treatment, P*k* is the effect of the *k*th period, (τP)*ik* is the treatment–period interaction, and Є*ijk* is the random error.

## 3. Results

### 3.1. Caffeine, Tannin, and Antioxidant Compounds in the Diets

Table 2 shows the results for caffeine and tannins. The values were significantly different (*p* < 0.05) among the diets, where caffeine content showed an increasing trend in ECP_10_ to ECP_20_ (7.20 to 7.27 mg/g DM); however, the values were not different (*p* > 0.05). For the tannins, a decrease was observed in EC_P0_ to ECP_10_ (*p* < 0.05) and an increase in ECP_10_ to ECP_20_ (*p* < 0.05).

The antioxidant capacity of the diets was not affected (*p* > 0.05) by the inclusion of ensiled coffee pulp (Table 2). Eight phenolic acids were identified (Table 2), and differences (*p* < 0.05) were found only for p-hydroxybenzoic acid, caffeic acid, syringic acid, vanillic acid, and p-coumaric acid, with an increment in ECP_0_, ECP_10_, and ECP_20_, respectively.

### 3.2. Performance of Sheep

No differences (*p* > 0.05) were found among treatments for DMI, DWG, FC, or WI, but an effect over time was observed (*p* < 0.05). Dry matter intake and WI increased over time, but DWG and FC values did not increase until after 45 days; after that time, the DWG decreased. All the productive variables were affected by time, but not the treatments or the interaction treatment × time (Table 3).

### 3.3. pH, Ammonia Nitrogen (NH_3_-N), and Volatile Fatty Acid (VFA)

The pH values were similar (*p* > 0.05) for all treatments on days 1 and 60. Regarding NH_3_-N, no differences (*p* > 0.05) were found among treatments or times (1 and 60 days), but differences were observed in the time–treatment interaction. The values for PropA and ButA in times and treatments were similar, whereas, for AcetA, differences were found in the time and in the interaction of time × treatment, but not for treatment (Table 4).

No differences (*p* > 0.05) in the concentration of FRAP were observed in the blood serum in ECP_0_ and ECP_10_; however, ECP_20_ was different (*p* < 0.05) at 1 and 60 days (Table 5). No differences (*p > *0.05) between treatments were observed for TBARS. No differences (*p* > 0.05) were observed in the nitrogen balance, total bacteria, and protozoa among the treatments (Table 5). The Inclusion of ECP did not affect (*p* > 0.05) the DMD, OMD, NDFD, or ADFD; however, the highest digestibility (*p* < 0.05) was observed with the inclusion of 10% ECP, whereas with 20% ECP, the digestibility was equal to that of the control treatment (Table 5). 

The addition of ECP to the diet did not have any effect (*p* > 0.05) on pH, slaughter weight, cold carcass weight (24 h postmortem), mesenteric fat, or carcass length. Hot and carcass weights were similar (*p* > 0.05) for hot and cold carcasses between treatments. However, differences (*p <* 0.05) were observed in the T° for the hot carcass. Meat composition was not affected (*p >* 0.05) by the inclusion of ECP in the diet (Table 6).

Adding ECP to the lambs’ diet did not impact the meat’s pH (*p* > 0.05). However, there was a decrease in pH from 24 h to 7 days of storage, regardless of the treatment (*p* < 0.05). The temperature at 7 days of storage was higher and affected the treatments, time (0 or 7 days), and treatment–time interaction (*p* < 0.05) (Table 7).

The L* and b* values were similar between treatments (*p* > 0.05) after 24 h and 7 days of storage. Redness (a*) increased in treatments with ECP (*p* > 0.05), while in ECP0, it decreased at 7 days of storage (Table 7). The inclusion of ECP in the diets did not affect WHC (*p* > 0.05) or texture after 24 h and 7 days of storage (Table 7).

There were no differences (*p* > 0.05) in FRAP between treatments, storage time (24 h or 7 days), or the interaction of treatment and time. The antioxidant capacity remained stable until day 7. The inclusion of ECP in the treatments did not affect the TBARS values (*p* > 0.05), except for the treatment with 20% of ECP, which showed the highest values at 24 h and 7 days of storage (*p* < 0.05). The meat oxidation trend increased as the storage time increased for all treatments (Table 7).

## 4. Discussion

Antioxidant capacity was equal in the three diets evaluated, possibly due to the similar content of phenolic compounds in the diets, such as chlorogenic acid, ferulic acid, and gallic acid, which have high antioxidant activity [34,35,36], or probably because the control diet contained the same proportion of sorghum as the diets with ensiled coffee pulp. High contents of bioactive phenolic compounds or condensed tannins, to which high antioxidant capacity has been attributed, have been reported in sorghum [37,38] and maize crop residues [39]. Araya et al. [40] mentioned that a higher antioxidant capacity does not always mean that its activity is better or more effective in vivo since it is the chemical structure that determines the absorption of polyphenols, and effectiveness in the organism depends on the bioavailability of these antioxidant compounds. Therefore, antioxidant capacity depends on the nature and concentration of the natural antioxidants in the diets.

Dry matter intake, DWG, FC, and WI did not significantly differ when ensiled coffee pulp was added to the diet. This result is concordant with a previous study by Vitto et al. [41]. These authors did not find differences when 15, 20, and 25% ensiled coffee pulp was included in the diet of sheep; the exception was DWG, which increased significantly compared to the control diet without ECP. Hernández-Bautista et al. [10] included 7, 14, 21, and 28% of ensiled coffee pulp in the sheep diet without affecting the productive parameters. Likewise, Salinas-Ríos et al. [9] included 8 and 16% ensiled coffee pulp and found no differences in the DMI, DWG, and FC.

In contrast, García et al. [42] and Souza et al. [43] added 15.23% coffee pulp and 10% coffee hulls to sheep diets and found no effect on the DMI or DWG. DM intake was not affected when coffee pulp was included at 10, 15, and 20% in the diet of lactating cows, according to Pedraza-Beltran et al. [44]. Salinas-Ríos et al. [9] mentioned that water consumption increases because of the caffeine in coffee pulp and that increasing the percentage of pulp in the diet increases the WI. However, moisture in the diet and environmental temperature could have affected the water intake because the experiment was conducted in the autumn when the temperature was low. It is well known that the diuretic effect of caffeine [45] increases urination frequency and N excretion [46]. It is possible that the amount of caffeine in the diet was not enough to increase N excretion in the urine, or the amount of tannin in the diet affected N excretion or productive performance [47]. Vargas et al. [48] fed coffee pulp to steers (0, 20, 40, and 60%) and did not find differences in N retention between the control treatment and the treatment with 20% coffee pulp. However, the retention and absorption of N decreased as the percentage of coffee pulp in the diet increased. Water intake significantly affects N retention; the lower the water intake, the higher the N retention because N is lost in the urine, and, thus, N retention is reduced [49]. WI was not significantly different between the control diet and the diets with ECP, which might be why no differences were observed in the N balance.

The addition and subsequent increase of ensiled coffee pulp in the diets did not significantly affect the pH, NH_3_-N, or the AcetA, PropA, and ButA concentrations. Teixeira et al. [50] found that coffee hulls did not affect the rumen pH when they made up 0, 7, 14, and 21% of the total DMI. Oliveira et al. [51] fed Holstein cows 40% concentrate containing 25% coffee hulls and found no effect on the rumen pH or the concentration of NH_3_-N. Krueger et al. [52] added commercial tannin extracts to the steers’ diets and found no effect on the rumen pH, the NH_3_-N concentration, or the molar proportions of AcetA, PropA, and ButA. Goiri et al. [53] report that adding coffee residues alters the interactions between bacterial taxa and VFA production, mainly those related to acetic and propionic acid production. The pH observed might be due to the rumen buffer capacity, including saliva activity, fiber fermentation, and VFA by the rumen epithelium [54], and not because of the inclusion of coffee pulp in the diet. Prabhudessai et al. [55] have cataloged caffeine as a microbial stimulant, suggesting its potential as a microbial fermentation enhancer. However, in a series of in vitro experiments, Portela [56] suggests that the concentration of VFA may affect coffee husk due to defaunation that affects VFA production and the acetate–propionate ratio.

The amount of caffeine and tannins in the diets did not harm the sheep’s rumen environment. Goiri et al. [53] mention that adding coffee residues alters the rumen’s bacterial structure and the relative amount of some bacterial taxa. High concentrations of tannins inhibit rumen bacteria, but only when they increase to more than 5% of the DM of the diet [57]. Tannins in the diets used in this study were within the range considered safe for the animals. Portela [56] cultured protozoa in vitro using coffee pulp and observed that coffee pulp did not decrease the concentration of the protozoa, and the pH was not affected. McSweeney et al. [58] noted that tannins inhibit bacterial growth. Andrade-Rivero et al. [59] report that 2% condensed tannins from quebracho (*Schinopsis balansae*) inhibit microbial growth, and a higher dosage is fatal for rumen microorganisms. Tannins also affect protozoa activity and reduce protozoa populations [7]. However, this effect depends on the type of tannins and the level of inclusion [60]. Animut et al. [61] demonstrated that high levels of tannins (50, 101, and 151 g/kg DM) reduce the number of protozoa in goats; in contrast, Jayanegara et al. [62] evaluated tropical plants with phenolic compounds and found no correlation between the amount of hydrolyzable and condensed tannins and the number of protozoa and bacteria.

The blood serum’s antioxidant capacity (FRAP) was the same for ECP_0_ and ECP_10_. However, it was different for ECP_20_. These results are similar to those of Kuskoski et al. [63], who evaluated the antioxidant capacity of commercial fruit pulps frozen by the ABTS and DPPH methods and found a high correlation between the phenolic content and the anthocyanins. The antioxidant capacity was higher in ECP_20_ because this treatment had the highest amount of phenolic acids, except for gallic acid. The antioxidant capacity of this treatment was possibly higher because the amount of ECP increased [64].

Bueno et al. [65] found a direct effect of tannin–protein complexes on fiber-digesting rumen bacteria, reducing cell-wall degradation. In this study, ensiled coffee pulp addition did not affect in vivo, possibly because the caffeine and the tannin in the diets were insufficient to affect nutrient digestibility. Differences among treatments were observed only in the CP digestibility; the highest values were found in the diet with 10% ECP. Souza et al. [66] found that up to 10% of coffee hulls can be included in the diet without decreasing the DM digestibility, OM, CP, NDF, total carbohydrates, nonfibrous carbohydrates, or total nutrients. However, Maldonado and Benezra [67], using coffee pulp in sheep diets, found differences in digestibility. Salinas-Ríos et al. [9] used 8 and 16% coffee pulp in sheep diets and found responses in the OM, CP, and NDF digestibility similar to those in our study.

Including ensiled coffee pulp did not affect the back-fat thickness and loin-eye area. These results differ from those of Poornahavandi and Zamiri [68], who found that when given 80 mg of caffeine and 100 mg of ephedrine to lambs, fat meat and internal fat decreased. When commercial tannins were given to steers, Krueger et al. [52] did not find any effect on the back-fat thickness and loin-eye area.

TBARS values were similar between treatments; therefore, ECP did not prevent the meat from oxidation at 7 days of storage. However, the oxidation process was slower compared with the results obtained by Salinas-Rios et al. [64], who mentioned values of 4.49, 4.36, and 3.55 nmol/mL for the control, 8, and 16%, respectively, of ECP in the diet. Similar results observed for TBARS between treatments might be due to the presence of chlorogenic and ferulic acid in the diets with ECP that can prevent lipid peroxidation due to their antioxidant capacity [3].

The pH values of the carcass at the time of slaughter and 24 h post mortem, in this study, were similar to those found by Reséndiz [69] (6.5 and 5.6) with alfalfa diets that contained antioxidants, and Ayala [70], which included tannins and vitamin E (6.00 y 5.6). Therefore, pH values are considered within the normal range [71]. The temperature differences observed in the hot carcass, being the highest with the diet containing 20% of ECP, can be attributed to the highest level of ECP inclusion in the diet. Majdoub-Mathlouthi et al. [72] mention that the level of concentrate inclusion and carcass weight does not affect pH but the temperature of the hot carcass.

The results obtained in this study are similar to those of Alberti et al. [73], who did not find differences in carcass weight and yield between treatments when vitamin E was given to calves, or Resendiz [69], with alfalfa diets high in tannins offered to lambs.

It was reported by Frutos et al. [74] that the weight of the empty gastrointestinal tract, the skin, and carcass fat deposits of finishing lambs differed between the control and those assigned to the treatment with hydrolyzable tannins. ECP inclusion in the diets in the present study did not affect carcass characteristics due to its antioxidant content. The different results observed might be due to antioxidant and forage type and the amount in the diet [75].

Humidity, protein, and ash values in the meat were higher than those observed by Salinas-Rios et al. [64], who included ECP in lambs’ diets. However, their study’s inclusion level was lower (8% y 16%) than in this research. They are similar to those reported by Resendiz [69] on humidity (77.3), protein (21.2), and ash (4.2%). This author included different amounts of alfalfa in the diets of Pelibuey lambs, which also contain antioxidants. Poornahavandi and Zamiri [68] reported increased protein in dry meat when 80 mg of caffeine and 8 mg of ephedrine were included; this effect was not observed in the present study or by Salinas-Rios et al. [9].

There were no differences in pH between treatments; although, at 7 days of storage, the pH decreased for all treatments. Zhang et al. [76] did not find differences in pH in vacuum-packed meat and kept it at 4 °C with licorice extract as an antioxidant supplement. However, they found differences between storage times (0, 2, 4, 6, and 8 days). The pH reduction through time (24 h and 7 d) observed in this study might be explained by the conversion of glycogen to lactic acid by anaerobic glycolysis [77]. The pH values in the meat analyzed in this study were found to be higher than 6.0 at 24 h post mortem (0 days). According to Watanabe et al. [78], lamb-muscle pH values can be classified as normal (<5.8), intermediate (between 5.8 and 6.3), and high (>6.3). The pH values observed in this study were close to 6.3, which may have been due to low levels of glycogen in the muscle prior to slaughter [79], rather than the EPC present in the diet.

Meat temperature from animals fed with ECP was stable, which is essential because of the relationship between the meat’s shelf life and temperature [80], given that meat’s shelf life decreases if the temperature increases. 

The a* and b* indexes indicate the meat’s deterioration by changing the meat’s color from red to brown and the myoglobin concentration [81]. In the present study, lightness (L*) and yellowness (b*) remained stable, while redness (a*) values increased in treatments containing ensiled coffee pulp. Luciano et al. [82] found that when feeding sheep with fresh forage and diets rich in antioxidants, there was a trend in the carcass to have higher values for a* during storage time due to lower myoglobin oxidation, improving color stability, and better meat appearance. Du et al. [83] report higher values for a* in chicken thighs fed with 10% sorghum rich in tannins after 7 days of storage at 4 °C. Ayala [70] used *guazuma ulmifolia* foliage at different levels in lambs’ diets as a tannin source, L*, a*, and b* had differences between treatments; however, as storage time progressed, there were no differences compared to the initial value. ECP inclusion improved a* in the meat at 7 days of storage and did not affect b* and L*, because tannins maintain meat color stability and slow meat darkening [73,84].

Zhong et al. [85] mention that including antioxidants in the diet can reduce drip loss, because they also regulate protein degradation by increasing calpain activity, which is associated with drip loss [86,87]. However, WHC was not different between treatments or time until 7 d of storage. The pH values in the meat analyzed in this study were found to be higher than 6.0 at 24 h post mortem (0 days). WHC of meat is affected by its pH level and the changes that occur in muscle proteins during handling and preservation [88]. In a study by Bezerra et al. [89], sheep meat was found to have a WHC ranging from 59.86 to 63.41 mL/100 g, which is higher than the values obtained in the present study. The difference in WHC values between the two studies could be due to the higher pH level observed in the sheep meat 24 hours post-mortem [90]. In a study by Zhang et al. [76] it was found that using licorice extracts as an antioxidant supplement did not have a significant effect on WHC in ovines. Similar results were observed by Reséndiz [68] when alfalfa was used in Pelibuey diets.

Meat shear force was similar between treatments with no effect due to storage time. These results coincide with Alberti et al. [73] when using antioxidants, flavonoids, and vitamin E in calves’ diets, and Resendiz [69], but differ from Moran et al. [91], who report a lower meat shear force in lambs’ meat from animals fed carnosic acid and vitamin E. However, age and weight at slaughter influence meat shear force because young animals have less connective tissue [92,93].

FRAP levels were stable during the 7 days of storage in all treatments, perhaps because all diets contained the same antioxidant compounds except the control diet that did not have caffeic acid. Although phenolic acids were not present in the same proportion in all diets, they might have influenced the FRAP and antioxidant capacity of the meat. The antioxidant capacity of a diet depends not just on one of its components but on all of its antioxidant components and the environment where they are, interacting with synergic or inhibitory effects [94,95,96]. The rumen environment might be affected by the FRAP results of this study. In vivo studies have shown adaptation to oxidative stress [63,97], which might affect carcass characteristics and meat oxidation. Moran et al. [91] found that feeding lambs with vitamin E decreased TBARS compared to the *Longissimus lumborum* and *Gluteus medius* control group at different storage times (0, 7, and 14 d).

## 5. Conclusions

The study showed that adding up to 20% of ensiled coffee pulp to the diets of fattening lambs did not affect the tannin and caffeine concentrations, dry matter intake, water intake, growth performance, rumen variables, or nitrogen balance. However, it did influence the antioxidant compounds in the diets, the antioxidant capacity in blood serum, and the digestibility of crude protein. The use of ensiled coffee pulp did not have any negative effects on the carcass or meat characteristics of the lambs, such as texture, water-holding capacity, or meat oxidation. However, after 7 days of meat storage, there was an increase in redness (a*), while lightness (L*) and yellowness (b*) remained constant. The study concluded that incorporating up to 20% of ensiled coffee pulp in Pelibuey lambs’ diets is possible without affecting their productivity, ruminal fermentation, nutrient digestibility, or carcass and meat characteristics.

## Figures and Tables

**Table 1:** Ingredients and chemical composition of diets fed to Pelibuey lambs.

Items	Treatments		
	ECP_0_	ECP_10_	ECP_20_
Ingredients (%)			
Soybean meal	21	22	20
Ground sorghum	39	38	40
Hay corn	35	25	15
Molasses	3	3	3
Mineral supplement *	2	2	2
Ensiled coffee pulp	0	10	20
Chemical composition on a dry basis (%)			
Dry matter	88.5	88.2	87.0
Organic matter	81.2	80.2	80.0
Crude protein	15.1	16.0	16.4
Neutral detergent fiber	22.2	23.4	25.1
Acid detergent fiber	37.9	36.9	38.9
Ether extract	2.2	2.9	2.4
Ash	8.2	7.4	8.2
Energy (Kcal/kg DM)			
Digestible energy	2878.59	3004.96	2846.93
Metabolizable energy	2360.45	2464.07	2334.48

ECP_0_: control diet; ECP_10_ and ECP_20_: 10 and 20% of ensiled coffee pulp in the diet, respectively; DM: dry matter; * CoSO_4_ 0.068%, CuSo_4_ 1.04%, FeSO_4_, 3.57%, ZnO 1.24%, MnSO_4_ 1.07%; IK 0.052%; y NaCl 92.96%.

**Table 2:** Caffeine, tannin, antioxidant compounds, and antioxidant capacity in the diet.

Item	Treatments			SEM	*p*-Value
	ECP_0_	ECP_10_	ECP_20_		
Caffeine (mg/g DM)	0.00 ^b^	7.20 ^a^	7.27 ^a^	0.105	<0.0001
Tannins (mg/g DM)	3.42 ^a^	2.06 ^c^	2.99 ^b^	0.011	<0.0001
Antioxidant capacity					
FRAP (nmol Trolox/mL)	1396.68	1386.18	1415.07	41.92	0.8896
Antioxidant compounds (mg/g DM)					
p-hydroxybenzoic acid	0.23 ^c^	0.55 ^b^	1.15 ^a^	0.029	0.0004
Chlorogenic acid	5.27	7.34	14.98	3.94	0.3242
Ferulic acid	0.95	0.98	1.06	0.018	0.0540
Caffeic acid	0.00 ^c^	0.65 ^b^	1.32 ^a^	0.008	<0.0001
Syringic acid	0.12 ^c^	0.34 ^b^	0.57 ^a^	0.022	0.0018
Gallic acid	0.09	0.53	0.47	0.212	0.3994
Vanillic acid	0.19 ^c^	0.44 ^b^	0.71 ^a^	0.024	0.0015
p-cumaric acid	0.18 ^b^	0.28 ^ab^	0.33 ^a^	0.018	0.0228

ECP_0_: control diet; ECP_10_ and ECP_20_: 10 and 20% of ensiled coffee pulp in the diet, respectively; DM: dry matter; SEM: standard error of the mean; FRAP: ferric reducing antioxidant power; ^a,b,c^ Means in the same row with different superscripts are significantly different (*p* < 0.05).

**Table 3:** Productive Performance, feed conversion, and water intake in Pelibuey lambs fed with different levels of ensiled coffee pulp.

Item	15 Days				30 Days				45 Days				60 Days				Treat	Per	Treat × Per
	0%	10%	20%	SEM	0%	10%	20%	SEM	0%	10%	20%	SEM	0%	10%	20%	SEM			
DMI (kg)	1.20 ^ax^	1.20 ^ax^	1.20 ^ax^	0.007	1.35 ^ay^	1.35 ^ay^	1.35 ^ay^	0.007	1.48 ^az^	1.49 ^az^	1.46 ^az^	0.007	1.53 ^aw^	1.54 ^aw^	1.51 ^aw^	0.006	0.2622	<0.0001	0.7489
DWG (g/d)	186.18 ^ax^	192.50 ^ax^	181.25 ^ax^	10.81	196.82 ^ay^	208.25^ay^	200.87 ^ay^	10.81	234.91 ^az^	225.25 ^az^	226.00 ^az^	10.81	190.73 ^aw^	190.62 ^aw^	181.87 ^aw^	10.81	0.9071	0.0202	0.9978
FC	6.86 ^ax^	7.21 ^ax^	7.36 ^ax^	0.36	7.44 ^ay^	6.92	6.85 ^ay^	0.36	6.79 ^az^	7.02 ^az^	6.47 ^az^	0.36	8.13 ^aw^	8.27 ^aw^	8.85 ^aw^	0.36	0.9881	0.0045	0.8808
WI (L/d)	2.63 ^ax^	2.54 ^ax^	1.82 ^ax^	0.09	2.99 ^ay^	2.84	2.56 ^ay^	0.09	3.35 ^az^	3.20 ^az^	3.01 ^az^	0.09	3.43 ^aw^	3.17 ^aw^	3.19 ^aw^	0.09	0.0884	<0.0001	0.0807

Ensiled coffee pulp in the diet of 0, 10, and 20%; DMI: dry matter intake; DWG: daily weight gain; FC: feed conversion; WI: water intake; SEM: standard error of the mean. ^a,x,y,z,w^ Means in the same row with different superscripts are significantly different (*p* < 0.05).

**Table 4:** Ruminal variables, loin eye area, and backfat thickness in Pelibuey lambs fed with different levels of ensiled coffee pulp.

Item	30 Days				60 Days				Treat	Per	Treat × Per
	ECP_0_	ECP_10_	ECP_20_	SEM	ECP_0_	ECP_10_	ECP_20_	SEM			
Rumen pH	6.37	6.30	6.37	0.03	6.40	6.42	6.28	0.03	0.6870	0.5754	0.1754
NH_3_-N (mg/dL)	16.48	13.61	16.57	0.99	17.17	16.23	9.58	0.99	0.1586	0.3340	0.0095
Volatile acid fatty (mmol/L)											
Acetic acid	40.63 ^ax^	42.80 ^ax^	39.20 ^ax^	1.65	22.26 ^ay^	26.26 ^ay^	36.56 ^ay^	1.65	0.0892	<0.0001	0.0153
Propionic acid	13.36	12.16	10.20	0.55	12.19	12.07	12.28	0.55	0.3196	0.7112	0.2069
Butyric acid	7.16	7.50	6.73	0.36	6.30	6.92	6.19	0.36	0.5586	0.1717	0.9533
Backfat thickness (mm)	2 ^ax^	2 ^ax^	1 ^ax^	0.06	2 ^ay^	2 ^ay^	2 ^ay^	0.1	0.113	<0.0001	0.1953
Loin eye area (cm^2^)	679.82 ^ax^	707.58 ^ax^	650.83 ^ax^	28.46	826.73 ^ax^	833.17 ^ax^	771.00 ^ax^	28.46	0.499	<0.0001	0.4416

ECP_0_: control diet; ECP_10_ and ECP_20_: 10 and 20% of ensiled coffee pulp in the diet, respectively; SEM: standard error of the mean; NH_3_-N: ammoniacal nitrogen; ^a,x,y^ Means in the same row with different superscripts are significantly different (*p* < 0.05).

**Table 5:** Antioxidant activity, nitrogen retention, concentration of total ruminal bacteria and protozoa, and in vivo digestibility in Pelibuey lambs fed with different levels of ensiled coffee pulp.

Item	Treatments				SEM	*p*-Value
	IV	ECP_0_	ECP_10_	ECP_20_		
Antioxidant activity in blood serum						
FRAP (nmol Trolox/mL)	415.00 ^b^	457.65 ^ab^	459.08 ^ab^	519.06 ^a^	21.99	0.0075
TBARS (nmol MDA/mL)	4.160	5.643	6.294	10.442	1.80	0.0990
Retained nitrogen (%)		8.498	9.362	7.987	3.370	0.1955
Rumen microorganisms						
Total bacteria (1 × 10^9^/mL)		23.68	23.57	23.50	0.310	0.1522
Protozoa (1 × 10^4^/mL)		13.33	14.20	14.14	0.064	0.1007
In vivo digestibility (%)						
Dry matter		69.99	72.95	70.68	2.33	0.6378
Organic matter		76.33	77.20	72.43	2.12	0.2874
Crude Protein		67.13 ^b^	74.99 ^a^	60.40 ^b^	2.36	0.0042
Neutral detergent fiber		62.41	64.57	63.45	2.07	0.7612
Acid detergent fiber		58.75	47.51	51.28	3.13	0.0754

IV: initial value; ECP_0_: control diet; ECP_10_ and ECP_20_: 10 and 20% of ensiled coffee pulp in the diet, respectively; SEM: standard error of the mean; FRAP: ferric reducing antioxidant power; TBARS: thiobarbituric acid reactive substances; MDA: malondialdehyd5e; ^a,b^ Means in the same row with different superscripts are significantly different (*p* < 0.05).

**Table 6:** Characteristics of the carcass and chemical composition of meat of Pelibuey sheep fed with different levels of ensiled coffee pulp.

Item	Treatments			SEM	*p*-Value
	ECP_0_	ECP_10_	ECP_20_		
Slaughtering data					
Fasting body weight (kg)	36.07	35.20	33.72	0.871	0.2247
Hot carcass weight (kg)	17.70	17.32	16.03	0.544	0.1386
Cold carcass weight (kg)	17.07	16.76	15.52	0.497	0.1251
Mesenteric fat (kg)	0.55	0.50	0.19	0.139	0.1955
pH (slaughter)	6.27	6.21	6.32	0.113	0.7757
pH_24h_	6.02	5.59	5.43	0.235	0.2488
Hot carcass temperature (°C)	18.05 ^b^	18.56 ^a^	18.32 ^ab^	0.111	0.0250
Cold carcass temperature (°C)	12.52	14.22	12.40	0.598	0.0896
Carcass length (cm)	57.5	56.00	55.62	1.160	0.5222
Yields (%)					
Hot carcass yield	49.00	49.18	47.54	0.195	0.4460
Cold carcass yield	47.35	47.60	46.03	0.918	0.4611
Chemical composition of meat					
Moisture	74.16	73.72	75.22	1.411	0.7433
Protein	21.52	21.85	22.15	0.201	0.1266
Ash	4.23	4.05	4.39	0.135	0.2144

ECP_0_: control diet; ECP_10_ and ECP_20_: 10 and 20% of ensiled coffee pulp in the diet, respectively; SEM: standard error of the mean; ^a,b^ Means in the same row with different superscripts are significantly different (*p* < 0.05).

**Table 7:** Meat characteristics of Pelibuey sheep fed with ensiled coffee pulp.

Item	24 h				7 d				Significance		
	ECP_0_	ECP_10_	ECP_20_	SEM	ECP_0_	ECP_10_	ECP_20_	SEM	Treat	Per	Treat × Per
pH	6.27 ^ax^	6.17 ^ax^	6.30 ^ax^	0.18	6.00 ^ay^	5.57 ^ay^	5.46 ^ay^	0.18	0.2596	0.0007	0.2927
Temperature (°C)	17.97 ^ax^	18.55 ^ax^	18.32 ^ax^	0.25	21.81 ^ay^	20.98 ^ay^	19.81 ^by^	0.25	0.0285	<0.0001	0.0019
Color											
Lightness (L*)	37.72	38.93	39.57	1.69	36.95	39.78	43.56	1.69	0.2013	0.1152	0.0971
Rednes*s* (a*)	20.70	20.16	20.83	0.63	19.50 ^b^	21.26 ^ab^	23.49 ^a^	0.63	0.0299	0.0988	0.0266
Yellowness (b*)	6.04	5.00	4.91	19.6	3.52	4.98	7.71	19.6	0.2965	0.9047	0.0675
WHC (mL/100 g)	61.08	46.13	42.86	19.94	56.19	26.36	35.97	19.94	0.6698	0.0952	0.5112
Texture (g/cm^2^)	2388.28	2597.63	2483.40	339.53	2668.01	2307.50	2675.39	339.53	0.9412	0.7841	0.4990
Antioxidant activity											
FRAP	31.47	31.75	33.66	7.70	39.08	43.96	46.22	7.70	0.8331	0.0910	0.9360
TBARS	0.41 ^ax^	0.47 ^ax^	0.52 ^ax^	0.10	4.74 ^ay^	4.23 ^ay^	5.18 ^ay^	2.66	0.9635	0.0162	0.9668

ECP_0_: control diet; ECP_10_ and ECP_20_: 10 and 20% of ensiled coffee pulp in the diet, respectively; SEM: standard error of the mean; WHC: water holding capacity; FRAP: ferric reducing antioxidant power; TBARS: thiobarbituric acid reactive substances; ^a,b,x,y^ Means in the same row with different superscripts are significantly different (*p* < 0.05).

## Data Availability

All data relevant to the study is included in the article.
